# The fallacy of coverage: uncovering disparities to improve immunization rates through evidence. Results from the Canadian International Immunization Initiative Phase 2 - Operational Research Grants

**DOI:** 10.1186/1472-698X-9-S1-S1

**Published:** 2009-10-14

**Authors:** Sharmila L Mhatre, Anne-Marie Schryer-Roy

**Affiliations:** 1 International Development Research Centre, 150 Kent Street, Ottawa, Canada; 2 AMS Consulting, PO 91-00606, Nairobi, Kenya

## Abstract

Immunization can and does save lives. However, the presence of vaccines does not easily translate into every child being vaccinated, and this is what the studies in this journal supplement reveal. From South Asia to West Africa,the evidence presented here reveals what we are calling the *fallacy of coverage*, going beyond uncovering the real vaccination rates to providing evidence on the reasons for the lack of effective coverage.

The evidence for the *fallacy of coverage* is part of an operational research program entitled the Canadian International Immunization Initiative Phase 2 (CIII2). Through a competitive peer review process, six research grants were awarded to increase access to and enhance immunization services. This journal supplement provides a forum for the presentation of the results of five of the six studies.

The story of the *fallacy of coverage* is made up of five theme areas of evidence - timeliness of immunization, social and gender inequities, vaccine efficacy, understanding demand side issues to tailor interventions, and national data sets masking actual district level coverage rates - that reveal the discrepancies in immunization coverage rates and the reasons behind these discrepancies. As part of the story, and to turn around the *fallacy of coverage*, the studies also provide proof of effective and locally relevant solutions.

Policies and funding, while keeping an eye on future diseases, clearly need to maintain and increase support to address *existing* vaccine-preventable diseases to increase coverage such that by 2015 we can achieve 90% national vaccination coverage and reach the MDG of reducing mortality rates among children under five by two-thirds.The results from the operational research grants of the CIII2 offer some answers on how to reach this goal by demonstrating how locally generated evidence can inform immunization strategies to ensure that children who need to get vaccinated will get vaccinated, and vaccinated on time.

## Introduction

Immunization can and does save lives. With existing vaccines, it is estimated that between two and three million deaths from diphtheria, tetanus, pertussis and measles are prevented annually [1]. There is a strong global recognition that increasing immunization coverage is an essential step towards reducing child mortality, the fourth Millennium Development Goal (MDG). Currently as a response, the chief goal set by UNICEF and WHO is to, by 2015 or earlier, reduce illness and death due to vaccine-preventable diseases by at least two thirds compared to 2000 levels.

Examination of the top three vaccine-preventable diseases in all regions of the world reveals the importance of focusing efforts on increasing coverage of existing vaccines (See Table 1). However, the presence of vaccines does not easily translate into every child being vaccinated, and this is what the studies in this journal supplement reveal. While progress is being made with global agencies reporting coverage rates of 81% for infants receiving three doses of diphtheria, pertussis and tetanus (DPT3) [2] and measles vaccination reaching 80% [3], the articles in this journal supplement reveal great disparities among and within countries. As such, from South Asia to West Africa the evidence presented here reveals what we are calling the *fallacy of coverage*. The studies in this supplement go beyond uncovering the real vaccination rates to providing evidence on the reasons for the lack of effective coverage. Some go as far as introducing locally-relevant interventions that actually improve the coverage rates of measles and DPT3 [4,5].

**Table 1:** Top vaccine-preventable diseases by region

Region	Diseases
South Asia	Measles,Tetanus,Pertussis
South-East Asia	Pertussis, Measles, Hep B
Latin America & Caribbean	Pertussis,Tetanus,Hep B
Sub-Saharan Africa	Measles,Pertussis,Tetanus

Source:http://www.worldbank.org/features/2006/tabledcpp.htm.

The evidence for the *fallacy of coverage* is part of an operational research program entitled the Canadian International Immunization Initiative Phase 2 (CIII2), initiated in September 2003. This initiative is a project of the Global Health Research Initiative (GHRI), which involves the collaboration of five major research/health agencies in Canada: International Development Research Centre (IDRC), Canadian International Development Agency (CIDA), Canadian Institutes of Health Research (CIHR), Health Canada (HC) and the Public Health Agency of Canada (PHAC). It is an important precedent of funding organizations coming together, pooling resources and strengths, to invest in filling the gaps in immunization research.

Through a competitive peer review process, six research grants were awarded to increase access to and enhance immunization services in CIDA's priority countries. This journal supplement in BioMed Central's open access journal *BMC International Health and Human Rights* provides a forum for the presentation of the results of five of the six studies.

The first paper that follows provides a global overview of the progress to date in immunization and some of the challenges from the CIII2 partners. It discusses the various approaches used by WHO and UNICEF to improve immunization coverage and at the same time sets the stage for the research results of the CIII2 operational research grants by impressing on the need for evidence to realise the full potential of immunization "in achieving the health-related MDGs"[6].

The subsequent 13 peer-reviewed papers unravel the *fallacy of coverage* and present the evidence for what is needed to get closer to achieving the fourth MDG. All the studies underline the importance of understanding the context in which the extent of immunization coverage is realised. Only through such operational and local research can we get the needed evidence to improve coverage - from South Asia to West Africa.

## Theme areas of evidence

The story of the *fallacy of coverage* is made up of five theme areas of evidence that reveals the discrepancies in immunization coverage rates and the reasons behind these discrepancies. It also includes evidence for turning around this *fallacy of coverage*. The first theme is the challenge of ***timeliness or age-appropriate immunization***. This is evident in the studies presented in this supplement from India [7], Pakistan [4,8], and Burkina Faso [9,10]. By assessing coverage through the analytical lens of age-appropriateness of coverage, these studies bring into question the progress of immunization coverage. In fact, as seen by the evidence, while overall coverage can be deceptively good, the story changes when one takes into account whether or not vaccines are administered on time. In India for example, while immunization coverage has overall increased, the work by Corsi *et al. *[7] shows that, nationally, complete age-appropriate coverage is still under 50%. Not only do age-appropriate immunization rates provide a truer picture of actual coverage, but such an approach is also useful for health workers and service providers - as noted in the work by Dugas *et al. *[9] and Bicaba *et al. *[10] - as it allows them to improve and tailor their immunization strategies to increase coverage. The implications for not providing immunization on time are reduced benefit of the vaccine and increased mortality.

The critical importance of the timely immunization theme was recently detailed in a review of data from 45 low-income and middle-income countries published in the *Lancet*[11].

The next key theme area of evidence to better understand the *fallacy of coverage* is the issue of ***social and gender equity***. Work by Corsi *et al. *[7] demonstrates that the progress of immunization coverage in India is hindered by the persistence of *gender inequities* across all socio-economic levels, resulting in girls having significantly lower coverage rates for bacille Calmette-Guérin (BCG), oral polio vaccine (OPV), DPT and measles vaccination. Interestingly, gender inequities affect who gets immunized or not, but does not affect the decision to immunize on time. Gender also needs to be considered in targeting interventions, as demonstrated through an in-depth ethnographic study by Dugas *et al. *[9] in Burkina Faso. The researchers found that in some communities despite the father's decision to vaccinate the children, mothers do not always bring them to be immunized. This gap between decision-making and actual vaccination practice requires interventions that target both parents.

The linkage between poor coverage and increasing inequities is demonstrated in the studies in Pakistan and Burkina Faso. Mitchell *et al. *[8] provide evidence on how ***poor access and mother's education*** (in urban areas only) were the key equity factors obstructing measles vaccination uptake in the Lasbela district of Pakistan. In the Nouna district of Burkina Faso, Sanou *et al. *[12] provide evidence for how the ***education of both parents*** along with the economic conditions of households affected immunization coverage. The authors did note, however, that the influence of economic conditions is complex as immunization services are free of charge, thus pointing to the importance of communication about the free services to avoid potential abuses by providers.

In addition to inappropriate coverage and increasing inequities linked to coverage, the Pakistan study uncovered the next key theme area, which is that of ***vaccine efficacy***. Through the development of a communication tool (a "balance sheet" summarizing published evidence on benefits and possible adverse effects of vaccination) to enable communities to balance costs and benefits of measles vaccination, Ledogar *et al *[13] uncovered a much *lower vaccine efficacy rate* in Lasbela, Pakistan, than expected. As such, the rate they found was 41.5% compared to the rate generally found in developed countries of 95% (range of 90-98%) [14]. Reasons for this discrepancy are discussed in the paper. While this "balance sheet" was not used in the randomised controlled trial of a community intervention in Lasbela, Pakistan, such a tool could serve as a web-based reference for project managers and health officials, helping them identify areas of improvement in immunization services.

Many of the articles in this supplement offer a strong demand side perspective, i.e. from existing and potential beneficiaries. This ***demand side evidence*** is the next key theme area which looks behind the discrepancies in vaccination rates to help tailor potential solutions. In the Pakistan series of articles, Shea *et al. *[15] conducted a systematic review of the literature on the impact of demand side interventions, demonstrating existing gaps and highlighting the need for such operational research. By focusing on the demand side, Dugas *et al. *[9] provide an increased understanding of why there is a lower than expected coverage rate in a health district in Burkina Faso. Their study points to the need to tailor interventions such that they take into account ***parents' perception of childhood illness*** and to the need to examine ***local vaccination procedures or requirements***. In this particular case, their research found that vaccination procedures served to deter rather than ensure access. In practice, immunization access was conditional on women going for antenatal care and acquiring and preserving a vaccination booklet for their child. In the same vein, Bicaba *et al. *[10] argue for the importance of understanding the ***reasons why some children are still not completely vaccinated***.

As part of a demand side analysis, understanding the local context is one of the keys to unravel the *fallacy of coverage*. Fourn *et al. *[16] in Benin ask the question: what are the factors that lead to reticence to vaccination among religious populations? Through the use of qualitative methods, their results suggest that ***interpretation of religious principles*** by church-going populations is primary in explaining reticence and that the solutions lie in ***creating an open dialogue among all actors***: reticent parents, their religious leaders and health authorities. Such an intervention is also alluded to by the work in Burkina Faso by Sanou *et al. *[12] and national EPI teams have used the results of the study.

The local level analyses also provide valuable information on existing immunization programs/campaigns. For example, Mitchell *et al. *[8] note that while other areas in Pakistan have demonstrated the positive impact of mobile vaccination teams, this was not the case in Lasbela district. Similarly, as seen in Burkina Faso, Haddad *et al*[17] note that Immunization Days did not have any impact on the performance of routine vaccination services. While both authors discuss this finding in their papers, it is worthy to note that these results from South Asia to West Africa further underscore that ***blueprint national programs/campaigns often do not resonate with communities*** as their assumptions are disconnected from local realities. Local data can be used to tailor such programs/campaigns to increase their effectiveness.

The studies in this supplement provided evidence from a local/district level and also compared their results to national data sets, thus presenting the last key theme area with regards to the *fallacy of coverage*. In the article by Cockroft *et al. *[18] the authors unravel a complex context where actual coverage rates are ***masked by national rates,*** and where there is ***heterogeneity in vaccination coverage*** between and within districts, and particularly between urban and rural areas in Pakistan. Despite this heterogeneity, as Cockroft *et al. *[18] point out, there is some ***commonality in the variables*** associated with vaccination uptake such as the quality of services, mother's education and knowledge of benefits. Local evidence on these issues can then be used to turn around the *fallacy of coverage*, thereby increasing immunization uptake.

## Evidence of locally relevant solutions

Beyond the five key theme areas of evidence that emerge from this supplement, the various studies also highlight effective and locally relevant solutions that can lead to increased immunization coverage rates. This was the case in Pakistan, where as detailed by Andersson *et al. *[4], researchers addressed the challenge of low measles coverage rates in Lasbela district by introducing an intervention involving an evidence-based and structured series of ***community discussions of vaccination's costs and benefits.*** The effect of this intervention was tested using a randomised controlled trial without relying on improved health services. In fact, through this operational research the team, working with communities and health workers, doubled the odds of measles vaccination uptake (20% increase) and tripled the odds of completing full DPT vaccination (29% increase) for this district. The Pakistan study thus provides evidence on how to improve the demand side of vaccination uptake at a relatively low cost (US$9 per child). Interestingly, the work in Mali by Koumaré *et al. *[19] and in Burkina Faso by Sanou *et al. *[12] similarly underscore the need to give priority to *providing information to communities* on the goals of immunization as well as the importance of tailoring interventions to local realities to improve immunization uptake.

The evidence also points to the fact that improving coverage rates requires work not only on the demand side, but also on ***the supply side***. As noted by Andersson *et al. *[4] in discussing the low efficacy rate of measles vaccine in Lasbela, Pakistan, improving service quality is needed to improve efficacy rates. As part of improving supply, the article by Djibuti *et al. *[20] focuses on health care providers and immunization managers in Georgia, documenting the effects of "supportive" supervision on the performance of the immunization program at the district level. Not surprisingly, within a framework of national immunization programs, such provider-based interventions can have a positive effect on coverage.

Finally, the last piece of evidence to help turn around the *fallacy of coverage* comes from the work by Haddad *et al. *[17], which focuses on ***system-related factors*** to explain disparities in immunization coverage among districts in Burkina Faso. By looking at a combination of factors (such as donor support, staffing standards, local strategies, immunization days and leadership by the district medical officer (DMO)) and their interaction, the authors conclude that the key is the ***"human factor"*** and the ability of ***good leadership*** to create the conditions for good performance. Even with limited access to donor supported initiatives or the presence of seasonal epidemics, with strong and committed leadership districts can adapt and perform well. Local strategies, as also demonstrated in the other studies, are also important and become more effective when linked to strong DMO leadership.

## Concluding comments

The articles are organised according to geography and research teams, starting in India and ending in Burkina Faso. Though, clearly the evidence repeats itself from South Asia to West Africa. Through locally based operational research, the story of the *fallacy of coverage* is revealed. Timeliness of immunization, social and gender inequities, vaccine efficacy, understanding demand side issues to tailor interventions, and national data sets masking actual district level coverage rates are the key theme areas of evidence. As part of the story, and to turn around the *fallacy of coverage*, the studies also provide proof of effective and locally relevant solutions: introducing structured series of community discussions on the cost-benefit of immunization, the role of supportive supervision and the role of strong leadership.

And such is the story of the *fallacy of coverage*, from South Asia to West Africa. The challenge now is how to build on the strength of the results and translate the evidence to global and national policies, programs and funding. This journal supplement is one step in that direction. At the same time, and as detailed by Duclos *et al. *[6], funding for improving immunization focuses not only on increasing coverage, but also on future diseases and vaccines. While it is important to keep an eye on future diseases, there is strong evidence - to which this supplement contributes - for the need to maintain and increase support to address *existing* vaccine-preventable diseases. While overall improvements in global coverage rates are undoubtedly taking place, in 2002 alone, it is estimated that 1.5 million children in all age groups died from diseases preventable by vaccines currently recommended by WHO (excluding measles) [21]. In addition, disparities persist not only between countries and regions, but also within countries and districts. Thus, the question arises on how best to allocate funds to increase coverage such that by 2015 we can achieve 90% national vaccination coverage and reach the MDG of reducing mortality rates among children under five by two-thirds. The results from the operational research grants of the CIII2 offer some answers by demonstrating how locally generated evidence can inform immunization strategies to ensure that children who need to get vaccinated will get vaccinated, and vaccinated on time. We are making progress, however we still have a way to go.

## List of abbreviations used

MDG: Millennium Development Goal; DPT: Diphtheria, Pertussis and Tetanus; CIII2: Canadian International Immunization Initiative Phase 2; GHRI: Global Health Research Initiative; IDRC: International Development Research Centre; CIDA: Canadian International Development Agency; CIHR: Canadian Institutes of Health Research; HC: Health Canada; PHAC: Public Health Agency of Canada; BCG: Bacille Calmette-Guérin; OPV: Oral polio vaccine; DMO: District Medical Officer.

## Competing interests

The authors declare they have no conflicts of interest.

## References

[B1] World Health Organization WHO immunization work: 2006-07 highlights 2008

[B2] United Nation's Children's Fund Annual report of the Executive Director: progress and achievements against the medium-term strategic plan 2009

[B3] World Health Organization Global Immunization Strategy, Report by the Secretariat Sixty First World Health Assemble 2008 10.1186/1472-698X-9-S1-S8

[B4] Andersson N Cockroft A Ansari NM Omer K Baloch M Foster AH Shea B Evidence-based discussion increases childhood vaccination uptake: a randomised cluster controlled trial of knowledge translation in Pakistan BMC Int Health Hum Rights 2009 9 Suppl 1 S8 10.1186/1472-698X-9-S1-S13 19828066 PMC3226240

[B5] Koumaré AK Traore D Haidara F Sissoko F Traoré I Dramé S Sangaré K Diakité K Coulibaly B Togola B MaÏga A Evaluation of immunization coverage within the Expanded Program on Immunization in Kita Circle, Mali: a cross-sectional survey BMC Int Health Hum Rights 2009 9 Suppl 1 S13 10.1186/1472-698X-9-S1-S2 19828057 PMC3226232

[B6] Duclos P Okwo-Bele J-M Gacic-Dobo M Cherian T Global immunization: status, progress, challenges and future BMC Int Health Hum Rights 2009 9 Suppl 1 S2 10.1186/1472-698X-9-S1-S3 19828060 PMC2762311

[B7] Corsi DJ Bassani DG Kumar R Awasthi S Jotkar R Kaur N Jha P Gender inequity and age-appropriate immunization coverage in India from 1992 to 2006. BMC Int Health Hum Rights 2009 9 Suppl 1 S3 10.1186/1472-698X-9-S1-S7 19828061 PMC3226235

[B8] Mitchell S Andersson N Ansari NM Omer K Soberanis JL Cockcroft A Equity and vaccine uptake: a cross-sectional study of measles vaccination in Lasbela District, Pakistan BMC Int Health Hum Rights 2009 9 Suppl 1 S7 10.1186/1472-698X-9-S1-S9 19828065 PMC3226239

[B9] Dugas M DubÃ© E KouyatÃ© B Sanou A Bibeau G Portrait of a lengthy vaccination trajectory in Burkina Faso: from cultural acceptance of vaccines to actual immunization. BMC Int Health Hum Rights 2009 9 Suppl 1 S9 10.1186/1472-698X-9-S1-S12 19828067 PMC3226241

[B10] Bicaba A Haddad S Kabore M Taminy E Feletto M Fournier P Monitoring the performance of the Expanded Program on Immunization, the case of Burkina Faso BMC Int Health Hum Rights 2009 9 Suppl 1 S12 10.1186/1472-698X-9-S1-S10 19828056 PMC3226231

[B11] Clark A Sanderson C Timing of children's vaccinations in 45 low-income and middle-income countries: an analysis of survey data. Lancet 2009 373 1543 1549 10.1016/S0140-6736(09)60317-2 19303633

[B12] Sanou A Simboro S Kouyaté B Gugas M Graham J Bibeau G Assessment of factors associated with complete immunization coverage in children aged 12-23 months: a cross-sectional study in Nouna district, Burkina Faso. BMC Int Health Hum Rights 2009 9 Suppl 1 S10 10.1186/1472-698X-9-S1-S6 19828054 PMC2762310

[B13] Ledogar RJ Fleming J Andersson N Knowledge synthesis of benefits and adverse effects of measles vaccination: the Lasbela balance sheet. BMC Int Health Hum Rights 2009 9 Suppl 1 S6 10.1186/1472-698X-9-S1-S5 19828064 PMC3226238

[B14] Centre for Disease Control, National Immunization Program Measles and Measles Vaccine, Epidemiology and Prevention of Vaccine Preventable Diseases. 2007 http://cdc.goc/vaccines/ed/vpd2007/download/slides.Measles10br.ppt

[B15] Shea B Andersson N Henry D Increasing the demand for childhood vaccination in developing countries: a systematic review. BMC Int Health Hum Rights 2009 9 Suppl 1 S5 10.1186/1472-698X-9-S1-S14 19828063 PMC3226237

[B16] Fourn L Haddad S Fournier P Gansey R Determinants of parents' reticence toward vaccination in urban areas in Benin (West Africa). BMC Int Health Hum Rights 2009 9 Suppl 1 S14 10.1186/1472-698X-9-S1-S15 19828058 PMC3226233

[B17] Haddad S Bicaba A Feletto M Taminy E Kabore M Ouedraogo B Contreras G Larocque R Fournier P System-level determinants of immunization coverage disparities among health districts in Burkina Faso:a multiple case study. BMC Int Health Hum Rights 2009 9 Suppl 1 S15 10.1186/1472-698X-9-S1-S4 19828059 PMC3226234

[B18] Cockcroft A Andersson N Omer K Ansari NM Khan A Chaudhry UU Ansari U One size does not fit all: local determinants of measles vaccination in four districts of Pakistan. BMC Int Health Hum Rights 2009 9 Suppl 1 S4 10.1186/1472-698X-9-S1-S13 19828062 PMC3226236

[B19] Koumaré AK Traore D Haidara F Sissoko F Traoré I Dramé S Sangaré K Diakité K Coulibaly B Togola B MaÏga A Evaluation of immunization coverage within the Expanded Program on Immunization in Kita Circle, Mali: a cross-sectional survey. BMC Int Health Hum Rights 2009 9 Suppl 1 S13 10.1186/1472-698X-9-S1-S11 19828057 PMC3226232

[B20] Djibuti M Gotsadze G Zoidze A Mataradze G Esmail LC Kohler JC The role of supportive supervision on immunization program outcome - a randomized field trial from Georgia. BMC Int Health Hum Rights 2009 9 Suppl 1 S11 19828055 10.1186/1472-698X-9-S1-S11 PMC3226230

[B21] World Health Organization, United Nation's Children Fund Global Immunization Data. 2009 http://www.who.int/immunization/newsroom/GID_english.pdf

